# Inter‐annual and regional variability of agronomic and grain quality traits of major Korean wheat cultivars under multi‐environmental conditions

**DOI:** 10.1002/jsfa.70862

**Published:** 2026-07-05

**Authors:** Goeun Lee, Jinhee Park, Kyeong‐Min Kim, Chon‐Sik Kang, Sookjin Kim, Jeong‐Heui Lee, Hyun‐jin Jung

**Affiliations:** ^1^ Winter Crop Research Division National Institute of Crop and Food Science, Rural Development Administration Wanju Republic of Korea; ^2^ Planning and Coordination Division National Institute of Crop and Food Science, Rural Development Administration Wanju Republic of Korea

**Keywords:** wheat, multi‐environment trials, phenology, grain quality, climatic regulation, cultivar stability

## Abstract

**BACKGROUND:**

Wheat agronomic performance and grain quality vary substantially depending on environmental conditions. However, few studies have evaluated agronomic performance, grain quality stability, and growth stage‐specific climatic regulation together across representative Korean wheat‐production environments.

**RESULTS:**

Phenological traits and amylose content showed relatively high stability across multiple environmental conditions, whereas yield components and protein‐related quality traits exhibited pronounced environmental variability. Climatic gradients integrating radiation, temperature, and moisture structured environmental differences and were differentially associated with crop responses across growth stages. Early season climate primarily influenced phenological development, whereas climatic conditions during reproductive growth exerted the strongest control over yield formation and protein‐related traits. An integrated grain quality index further showed that environmental effects were reflected through changes in overall quality performance and differences in interannual stability.

**CONCLUSION:**

Variations in wheat performance and grain quality across different environments were governed by structured climatic gradients and developmental timing rather than by isolated site effects. This study provides an analytical framework for distinguishing stable and environment‐sensitive wheat traits by integrating multi‐environment field trials, growth stage‐specific climate gradients, and composite grain quality assessment under Korean agro‐climatic conditions. © 2026 The Author(s). *Journal of the Science of Food and Agriculture* published by John Wiley & Sons Ltd on behalf of Society of Chemical Industry.

## INTRODUCTION

Wheat (*Triticum aestivum* L.) is one of the world's most important cereal crops and a major source of calories and plant proteins for the global population.^1^, ^2^ Beyond its role as a staple food, wheat is an important industrial crop used to produce a wide range of end‐use products, such as bread, noodles, and processed foods, for which grain quality is a determinant of processing performance and consumer acceptance.^3^, ^4^ Consequently, modern wheat production systems must maintain stable yields and consistent grain quality.^5^, ^6^

In Korea, domestic wheat production has attracted increasing attention amid concerns about food security, climate change, and global supply chain instability.^7^ Although cultivation has expanded through national breeding and policy initiatives, production environments remain highly heterogeneous and are subject to pronounced inter‐annual climatic variability.^8^, ^9^, ^10^ These conditions challenge the achievement of stable agronomic performance and consistent grain quality across regions and years, highlighting the need for systematic evaluation of wheat cultivars across multiple environments.^11^

Wheat productivity and grain quality are governed by complex interactions between genetic potential and environmental conditions, particularly during critical developmental stages, such as heading and grain filling.^12^ Phenological traits influence exposure to favorable or adverse climatic conditions, whereas yield components and grain quality traits are sensitive to temperature, precipitation, and solar radiation.^13^, ^14^ Increasing climate variability and the more frequent occurrence of extreme weather events in East Asia have intensified environmental uncertainty, potentially accelerating crop development, shortening the grain‐filling period, and altering biomass partitioning and grain composition.^15^, ^16^, ^17^

Grain quality traits, including protein content, gluten content, and sodium dodecyl sulfate (SDS) sedimentation values, are particularly responsive to late‐season environmental conditions.^18^ Elevated temperatures during grain filling may enhance protein accumulation, whereas excessive precipitation may reduce grain quality by reducing radiation interception and increasing lodging risk.^19^, ^20^Other quality attributes, such as amylose and ash content, also exhibit environment‐dependent variation, indicating that grain quality reflects genotype–environment interactions rather than fixed genetic expression.^21^, ^22^, ^23^

Despite the importance of environmental regulations, integrated evaluations combining agronomic traits, yield components, grain quality traits, and climatic variables under Korean production conditions remain limited.^24^ ^,^ ^25^ Previous studies have often focused on specific traits, locations, or years, limiting generalizations across diverse agro‐climatic zones.^26^, ^27^ Trait stability and variability across environments have rarely been quantified explicitly, limiting the application of research results to cultivar deployment and management strategies.^28^, ^29^

Multi‐environment trials (METs) provide a robust framework for quantifying genotype–environment interactions and assessing trait stability and adaptability.^30^, ^31^Within this framework, the coefficient of variation (CV) provides an intuitive measure of trait variability and environmental sensitivity.^32^, ^33^ Integrating climatic data into MET analyses further enables the identification of climatic drivers underlying agronomic and quality responses and supports the growth stage‐specific interpretation of trait variability.^34^

The present study conducted a comprehensive multi‐environment evaluation of major winter wheat cultivars across contrasting agro‐climatic production environments. Field trials were performed at eight representative wheat‐growing locations during the 2022 and 2023 growing seasons using the widely grown cultivars Keumkang and Jokyoung. Agronomic traits, yield components, grain quality traits, and weather variables were integrated into a unified dataset to quantify interannual and regional trait variability, assess trait stability using the coefficient of variation, and characterize spatiotemporal performance patterns. Climatic regulation was examined through correlation analyses of whole‐season and growth‐stage–specific weather indices. Finally, an integrated grain quality assessment was conducted using a composite quality score to compare the overall grain quality potential across environments.

Unlike previous studies, which primarily evaluated mean environmental responses, this study integrated climatic ordination, developmental stage‐specific climate analyses, and trait stability assessment into a unified framework for explaining how structured climatic gradients are translated into trait‐specific agronomic and grain quality responses across environments. The framework provides a quantitative basis for improving yield stability, improving grain quality, and developing environment‐specific wheat production strategies under heterogeneous and changing agro‐climatic conditions.

## MATERIALS AND METHODS

### Experimental design and plant materials

**Figure 1:**
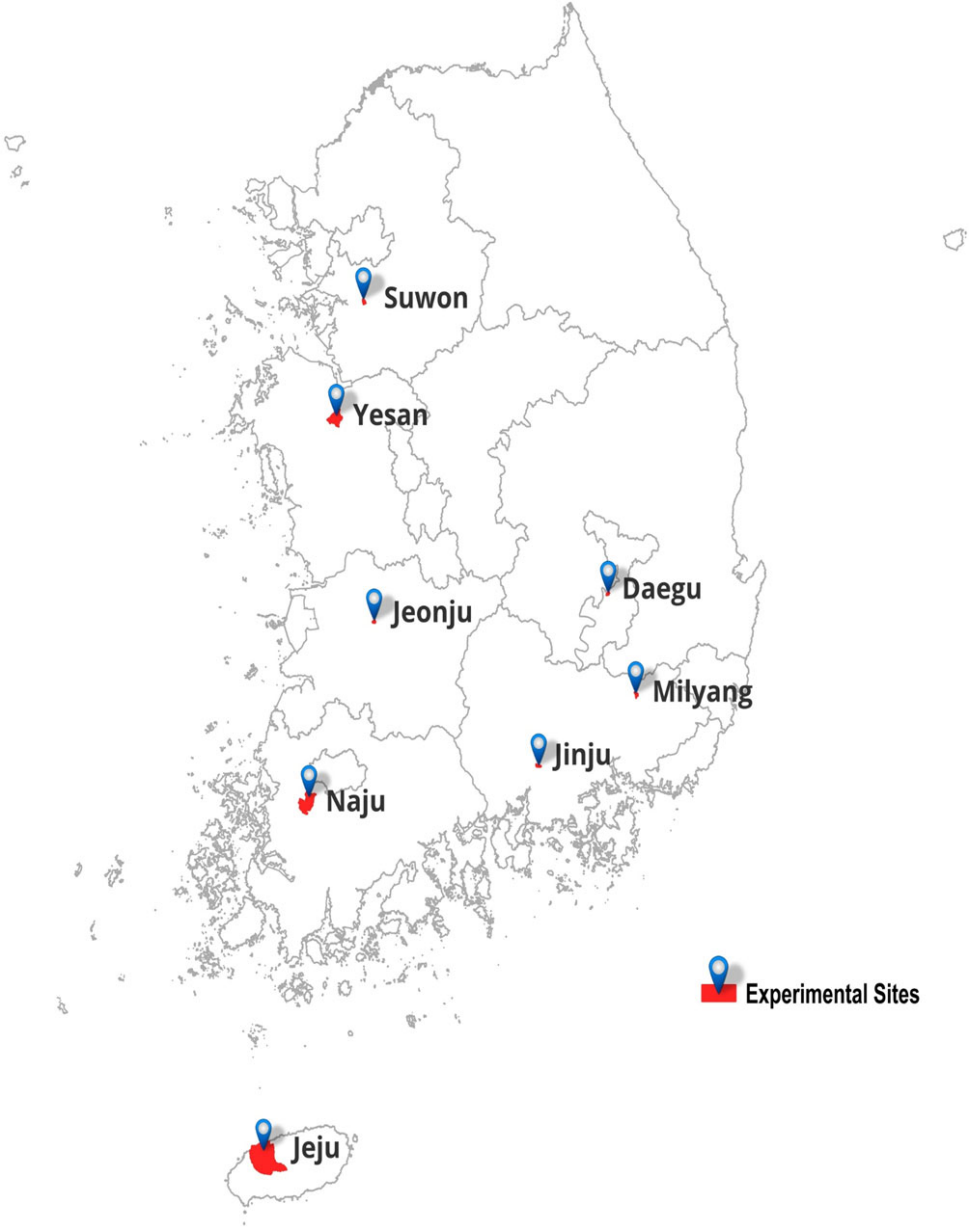
Locations of the eight experimental sites used for the multi‐environment wheat field trials conducted during the 2022 and 2023 growing seasons.

Multi‐environment field trials were conducted during the 2022 and 2023 growing seasons at eight representative wheat‐producing sites in Korea: Suwon, Yesan, Jeonju, Naju, Jinju, Milyang, Daegu, and Jeju (Fig. 1). These sites encompassed diverse agro‐climatic conditions and both upland and paddy–upland rotation systems. Cropping systems were considered as inherent components of each site‐specific production environment rather than independent experimental factors, and all sites were treated as independent environments.

Two widely cultivated winter wheat cultivars, ‘Jokyoung’ and ‘Keumkang’, were used as representative commercial cultivars.^35^ At each site, the experiments were arranged in a randomized complete block design with three replicates. Plot size was 9.0 m^2^ (6 m × 0.25 m × 6 rows) in upland fields and 7.5 m^2^ (5 m × 1.5 m) in paddy–upland rotation fields, reflecting local cultivation practices.

Sowing was performed according to site‐specific agronomic schedules, and standard management practices were applied at all sites. Seeding rate, row spacing, and fertilization regimes followed local recommendations, with basal and topdressing applications of nitrogen (N), phosphorus (P_2_O₅), and potassium (K_2_O) adjusted to local soil fertility and cropping conditions. Irrigation, weed control, and pest management were implemented according to standard local farming practices to ensure uniform crop establishment and growth.

Table 1 reports detailed information on site‐specific cultivation practices, cropping systems, and fertilization regimes.

**Table 1:** Site characteristics and management practices of multi‐environment wheat field trials conducted at eight locations in Korea during the 2022 and 2023 growing seasons

Location	Cropping system	Soil texture	Seeding rate (kg ha^−1^)	Plot size (m^2^)	Fertilization* (N–P–K) (kg ha^−1^)
Daegu	Upland	Loam	140	9	91–74–39 (52 N)
Jeju	Upland	Silt loam	160	9	44–90–36 (32 N)
Jeonju	Paddy–upland rotation	Clay loam	160	7.5	72–112–85 (98 N)
Jinju	Paddy–upland rotation	Silt loam	160	7.5	38–73–31 (38 N)
Milyang	Paddy–upland rotation	Clay loam	160	7.5	46–74–39 (45 N)
Naju	Paddy–upland rotation	Sandy loam	160	7.5	52–120–90 (78 N)
Suwon	Upland	Clay loam	140	9	55–74–39 (36.4 N)
Yesan	Upland	Loam	140	9	91–74–39 (52 N)

* Fertilization rates are expressed as the total seasonal application of N–P_2_O₅–K_2_O (kg ha^−1^). Values in parentheses indicate nitrogen applied as topdressing. Basal fertilizer was applied by broadcast incorporation prior to plowing, and topdressing nitrogen was applied once at the regrowth stage.

### Agronomic traits and yield measurement

Agronomic traits, yield components, and yield‐related parameters were evaluated throughout the growing season in accordance with standardized national investigation guidelines.^36^ Crop developmental timing was monitored in the field, and growth duration traits were derived from recorded phenological dates. Days to heading (DTH) was defined as the number of days from sowing to heading, the grain filling period (GFP) as the number of days from heading to maturity, and days to maturity (DTM) as the number of days from sowing to maturity. Heading was recorded when approximately 40% of the tillers within a plot had headed, and maturity was defined as the stage at which most spikes reached physiological maturity, indicated by complete spike yellowing.

Morphological traits were assessed using representative plant samples. Plant height (PH, cm) was measured from the soil surface to the spike neck of the tallest plants, and spike length (SL, cm) was measured from the spike neck to the spike tip, excluding the awns. The spike number per unit area (SPU, spikes m^−2^) was estimated by converting spike counts to a per m^2^ basis, and grains per spike (GPS, grains spike^−1^) were determined from representative spikes sampled from each plot.

At harvest, grain yield (GY, kg ha^−1^) was measured after threshing and drying, adjusted to a standard moisture content of 14%, and expressed on an area basis. The test weight (TW, g L^−1^) was determined using a 1 L volumetric tester with cleaned grain samples, and the thousand kernel weight (TKW, g) was measured by counting and weighing 1000 kernels. All measurements were conducted using standardized protocols to ensure consistency and reproducibility across environments.

### Grain quality analysis

Grain quality analyses were conducted according to methods approved by the American Association of Cereal Chemists (AACC).^37^ Protein content (g kg^−1^), gluten content (g kg^−1^), and index, amylose content (g kg^−1^), SDS sedimentation volume (mL), particle size distribution (PSI), and ash content (g kg^−1^) were measured using certified analytical instruments and internationally recognized protocols (Table 2).

**Table 2:** Summary of analytical methods, instruments, and American Association of Cereal Chemists (AACC) international standard protocols used for grain quality determination in multi‐environment wheat trials

Trait	Method	Instrument	Standard
Protein content	Dumas combustion method	Elementar nitrogen/protein analyzer (Elementar Analysensysteme GmbH, Langenselbold, Germany)	AACC 46‐30
Gluten content and index	Wet gluten washing and centrifugation	Glutomatic 2200 (Perten Instruments AB, Hägersten, Sweden)	AACC 38‐12
Amylose content	Iodine colorimetric method	Ultraviolet‐visible (UV‐visible) spectrophotometer	AACC 61‐03
SDS sedimentation volume	SDS–lactic acid sedimentation	100‐mL graduated sedimentation cylinder	AACC 56‐61
Particle size index (PSI)	Laser diffraction	LS 13 320 Laser Diffraction Particle Size Analyzer (Beckman Coulter Inc., Brea, CA, USA)	AACC 55‐40
Ash content	Dry ashing	Muffle furnace	AACC 08‐01

Abbreviations: AACC, American Association of Cereal Chemists; SDS, sodium dodecyl sulfate; PSI, particle size index; UV–Vis, ultraviolet–visible.

### Weather data and growth stage classification

Daily weather data were obtained from the synoptic meteorological observation network operated by the Korea Meteorological Administration, using records from the nearest official station for each experimental site. Climatic variables included daily air temperature, precipitation, sunshine duration, and solar radiation during the 2022 and 2023 wheat growing seasons.

The growing seasons were divided into three developmental periods: sowing to heading (SH), heading to maturity (HM), and sowing to maturity (SM). Climatic indicators were calculated for each period, including growing degree days (GDD) (base temperature 0 °C), mean air temperature (MT), diurnal temperature range (DTR), cumulative precipitation (CP), sunshine duration, and cumulative solar radiation (CSR). Temperature‐based indices were averaged over each growth stage, and precipitation, sunshine duration, and solar radiation were summed.

Because solar radiation observations were unavailable at the Milyang experimental site, CSR was excluded from the multivariate climatic ordination to maintain a complete climatic dataset across all site‐year environments. Sunshine duration, available for all sites, was used as a proxy for radiative conditions in the principal component analysis.

### Statistical analysis

Phenotypic variability in agronomic traits, yield components, and grain quality traits was quantified using the CV (%), which was calculated as the ratio of the standard deviation to the mean.^38^ Temporal and spatial variations across the years and experimental sites were examined using descriptive and distribution‐based analyses.

The stage‐specific climatic regulation of wheat traits was assessed by examining Pearson's correlations between climatic variables and phenotypic traits. Climatic indicators were aggregated for three developmental periods (SH, HM, and SM) and included GDD, MT, DTR, CP, sunshine duration, and CSR. Correlations involving CSR were calculated using only site–year records for which CSR data were available, whereas all other correlations were based on the full dataset.

The climatic structure of the production environments was characterized by principal component analysis (PCA) of standardized stage‐specific climatic variables. All variables were standardized to a mean of 0 and a variance of 1 before analysis. Because CSR data were unavailable for the Milyang experimental site, CSR was excluded from the PCA, and sunshine duration was used as a proxy for radiative conditions. The first two principal components were retained to represent the dominant climatic gradients.

The overall grain quality performance was evaluated using a composite grain quality index derived from the standardized Z‐scores of individual quality traits, including protein content, gluten content, amylose content, SDS sedimentation volume, PSI, and ash content. The Z‐scores were calculated as follows:

$$Z=\left(x-\mu \right)/\sigma$$

where x represents the observed trait value for a given environment, and μ and σ denote the mean and standard deviation of the corresponding trait across all environments.

The composite grain quality index was calculated as the arithmetic mean of the standardized trait scores:

$$\text{composite quality index}=\begin{array}{l} (Z_{\text{protein}}+Z_{\text{gluten}}+Z_{\text{amylose}}\\ +\;Z_{\mathrm{SDS}}+Z_{\mathrm{PSI}}+Z_{\mathrm{ash}})/6 \end{array}$$

allowing direct comparison of regional grain quality performance on a common scale.

All statistical analyses were performed using Python (version 3.10) software. Data processing and statistical computations were conducted using NumPy and Pandas. Visualization and exploratory analyses were performed using Matplotlib and Seaborn. Principal component analysis was performed using the *scikit‐learn* package. All analyses followed standardized and reproducible workflows to ensure consistency across datasets.

## RESULTS

### Trait variability across years and locations

**Figure 2:**
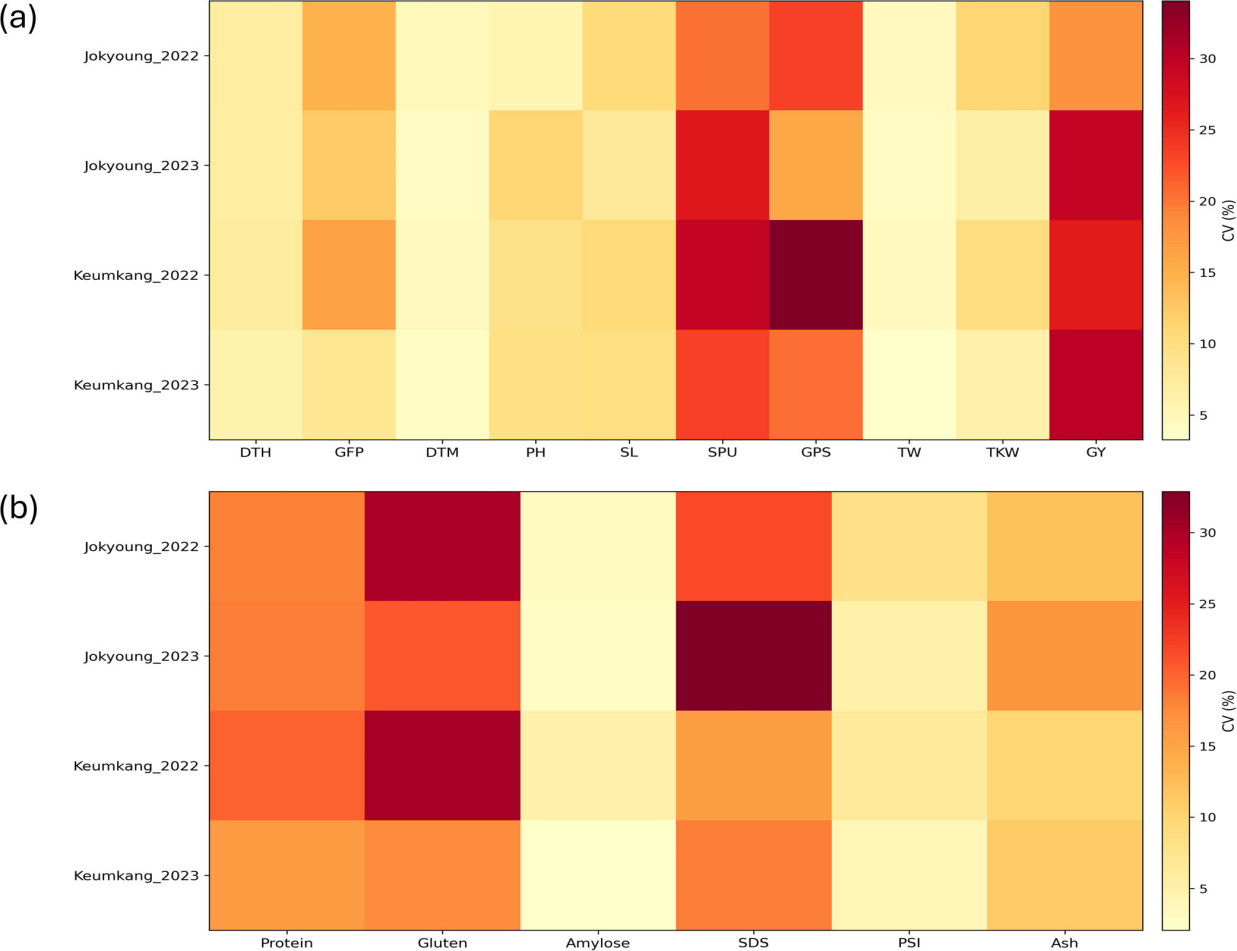
Cultivar‐year‐specific variability of agronomic traits, yield components, and grain quality traits, expressed as coefficients of variation (CV, %) for two winter wheat cultivars (‘Jokyoung’ and ‘Keumkang’), grown in 2022 and 2023: (a) agronomic traits and yield components, including days to heading (DTH), grain filling period (GFP), days to maturity (DTM), plant height (PH), spike length (SL), spikes per unit area (SPU), grains per spike (GPS), test weight (TW), thousand‐kernel weight (TKW), and grain yield (GY); and (b) grain quality traits, including protein content, gluten content, amylose content, sodium dodecyl sulfate (SDS) sedimentation value, particle size index (PSI), and ash content.

**Figure 3:**
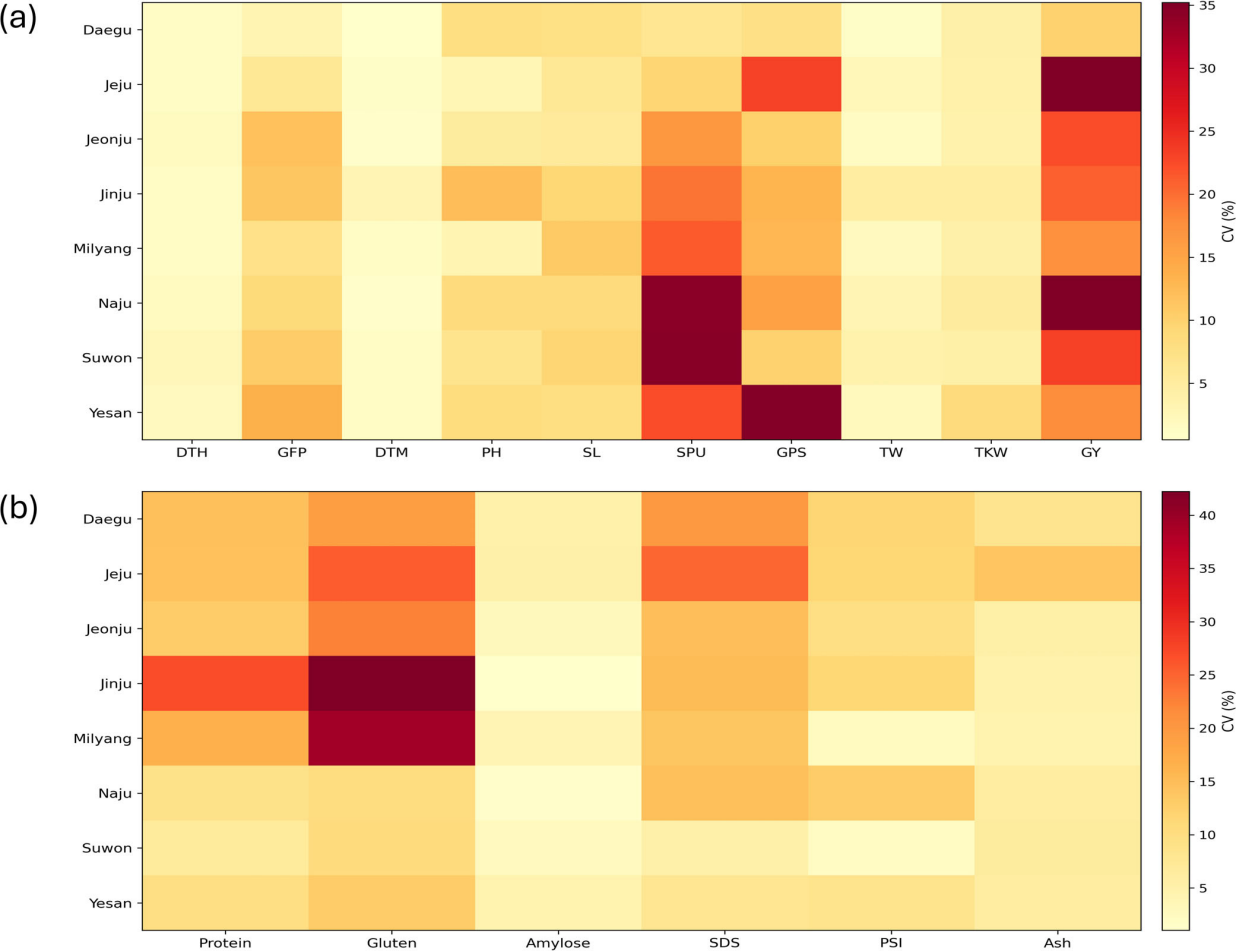
Site‐level variability of agronomic traits, yield components, and grain quality traits across eight experimental sites, expressed as coefficients of variation (CV, %): (a) agronomic traits and yield components, including days to heading (DTH), grain filling period (GFP), days to maturity (DTM), plant height (PH), spike length (SL), spikes per unit area (SPU), grains per spike (GPS), test weight (TW), thousand‐kernel weight (TKW), and grain yield (GY); and (b) grain quality traits, including protein content, gluten content, amylose content, sodium dodecyl sulfate (SDS) sedimentation value, particle size index (PSI), and ash content.

**Figure 4:**
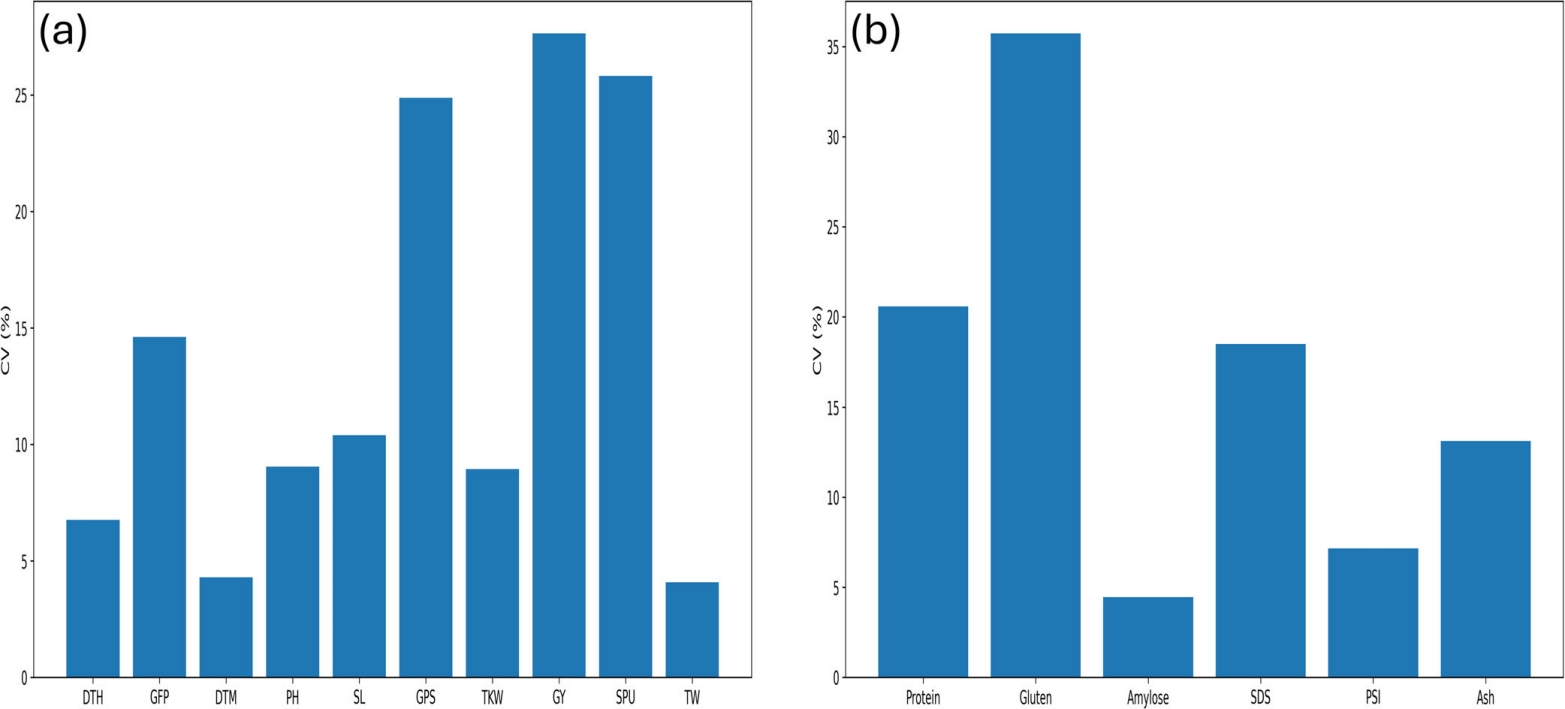
Overall phenotypic variability of agronomic traits, yield component, and grain quality traits across multi‐environment trials, expressed as coefficients of variation (CV, %): (a) agronomic traits and yield components, including days to heading (DTH), grain filling period (GFP), days to maturity (DTM), plant height (PH), spike length (SL), spikes per unit area (SPU), grains per spike (GPS), test weight (TW), thousand‐kernel weight (TKW), and grain yield (GY); and (b) grain quality traits, including protein content, gluten content, amylose content, sodium dodecyl sulfate (SDS) sedimentation value, particle size index (PSI), and ash content.

Phenotypic variability in agronomic traits, yield components, and grain quality traits was evaluated across multi‐environment wheat trials conducted during the 2022 and 2023 growing seasons using coefficients of variation (CV, %). Variability was assessed at three scales: cultivar–year combinations (Fig. 2), experimental locations (Fig. 3), and across all environments (Fig. 4). Table 3 presents summary statistics, including year‐specific means, standard deviations, and overall CV values.

**Table 3:** Descriptive statistics and phenotypic variability of agronomic traits, yield components, and grain quality traits across multi‐environment wheat trials in 2022 and 2023

Category	Traits	Descriptive statistics (2022)		Descriptive statistics (2023)		Stability (CV,%)
		Mean	SD	Mean	SD	
Phenology	DTH	172.7	12.2	170.3	11.0	6.8
	GFP	40.3	6.2	46.3	4.9	14.6
	DTM	213.0	9.7	216.6	8.4	4.3
Morphology	PH	76.0	5.8	75.4	7.8	9.0
	SL	8.3	0.9	8.4	0.8	10.4
Yield component	SPU	774.4	199.8	698.2	173.7	25.8
	GPS	30.0	8.7	28.9	5.7	24.9
	TW	818.6	35.8	813.1	30.8	4.1
	TKW	46.7	5.1	46.9	3.0	8.9
^a^ Grain yield	GY	5637.9	1343.7	4836.6	1450.5	27.6
^b^ Grain quality	Protein	112.9	24.3	124.3	23.5	20.6
	Gluten	99.3	35.8	106.2	23.2	29.3
	Amylose	244.4	11.0	256.7	7.2	4.4
	SDS	59.9	11.2	54.3	14.5	23.0
	PSI	72.2	5.2	84.3	3.7	9.7
	Ash	4.5	0.5	4.5	0.7	13.1

^a^ Grain yield was expressed as kg ha^−1^.

^b^ Protein, gluten, amylose, and ash contents are expressed as g kg^−1^. SDS, sodium dodecyl sulfate sedimentation volume (mL). PSI, particle size index.

Across the cultivar–year combinations, the phenological traits exhibited consistently low variability (Fig. 2(a)). Days to maturity and DTH showed the lowest CV values across cultivars and years, whereas GFP displayed moderate variability. Morphological traits, including PH and SL, also exhibited relatively low variability. These results indicate that developmental timing and basic plant architecture were comparatively stable across environments and years.

Yield‐related traits showed greater heterogeneity. Spike number per unit area, GPS, and GY showed the highest CV values across cultivar–year combinations. In contrast, TKW and TW exhibited comparatively low variability, suggesting greater stability of grain‐size‐related traits than of yield‐formation processes.

Grain quality traits showed contrasting stability patterns (Fig. 2(b)). The gluten content exhibited the highest variability, followed by the SDS sedimentation volume and protein content. In contrast, the amylose content consistently showed the lowest variability across cultivar–year combinations, whereas the PSI and ash content exhibited intermediate variability. The marked contrast between amylose content and protein‐related quality traits suggests that different grain quality components respond differently to environmental variation and should not be assumed to exhibit uniform environmental sensitivity.

The location‐specific analyses further highlighted the influence of environmental factors on trait variability (Fig. 3). Phenological traits remained relatively stable across the experimental sites. In contrast, yield‐related traits and protein‐associated quality traits, particularly gluten content, showed pronounced spatial variability. Amylose content remained consistently stable across all locations. This consistent stability across geographically distinct production environments further supports the view that amylose content is less environmentally responsive than protein‐associated grain quality traits.

Overall variability across all environments (Fig. 4) reinforced these patterns. Grain yield and major yield components exhibited the highest overall variability, whereas phenological traits and amylose content showed the highest stability. Importantly, the overall variability patterns revealed a clear separation between environmentally stable traits (DTH, DTM, amylose content, and TW) and environmentally responsive traits (GY, SPU, GPS, protein content, gluten content, and SDS sedimentation volume). These results indicate that phenological development and starch composition are relatively robust across environments, whereas yield components and protein‐related quality traits are highly sensitive to environmental variation. The contrasting stability patterns further demonstrate that environmental heterogeneity does not influence all agronomic and grain quality traits uniformly but instead affects specific components of wheat productivity and grain quality selectively.

### Temporal and spatial variability of growth and grain quality

**Figure 5:**
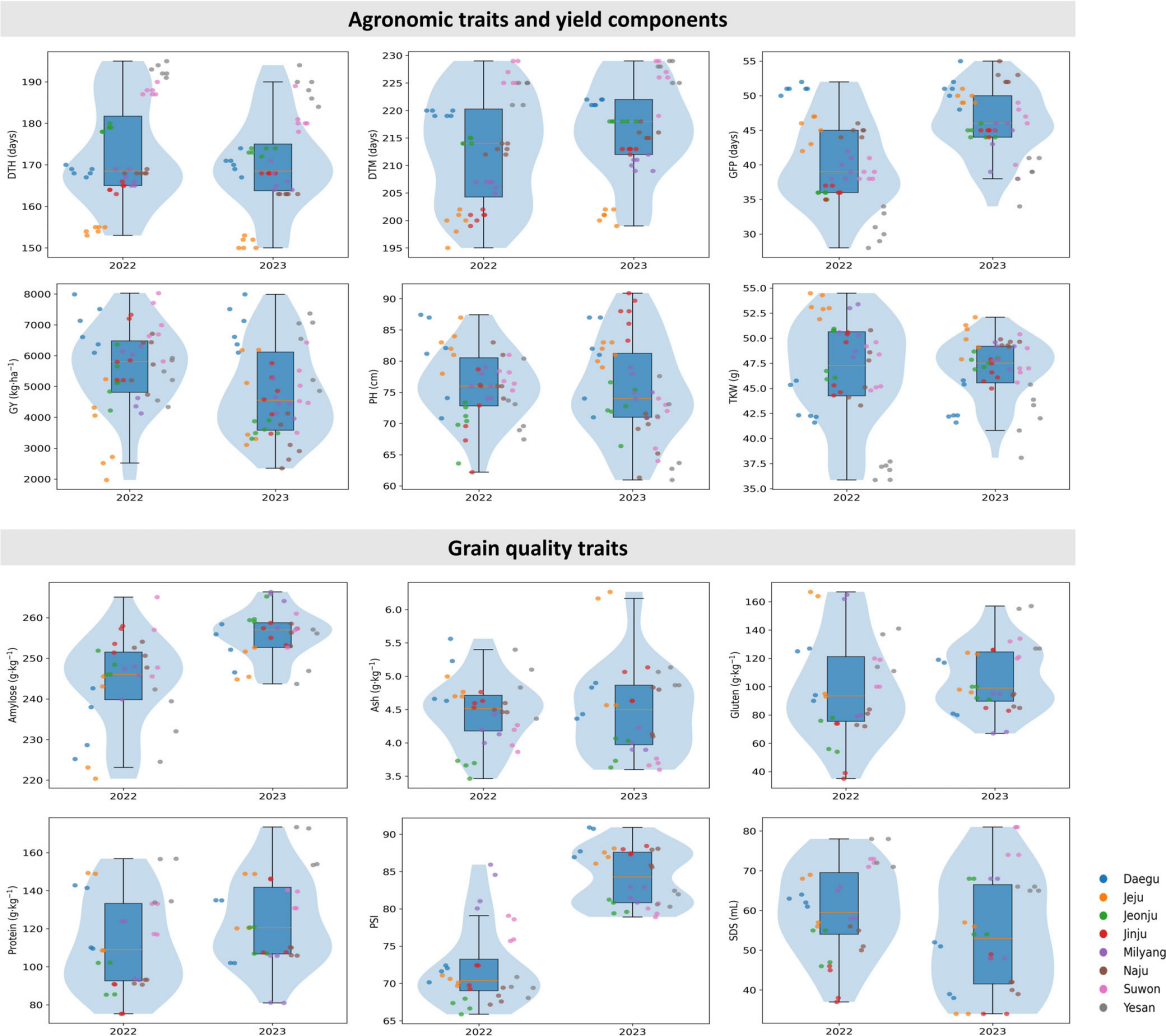
Inter‐annual distributions of agronomic traits, yield components, and grain quality traits observed across multi‐environment wheat trials in 2022 and 2023, showing year‐to‐year variation in agronomic and yield‐related traits (upper panels) and grain quality traits (lower panels).

**Figure 6:**
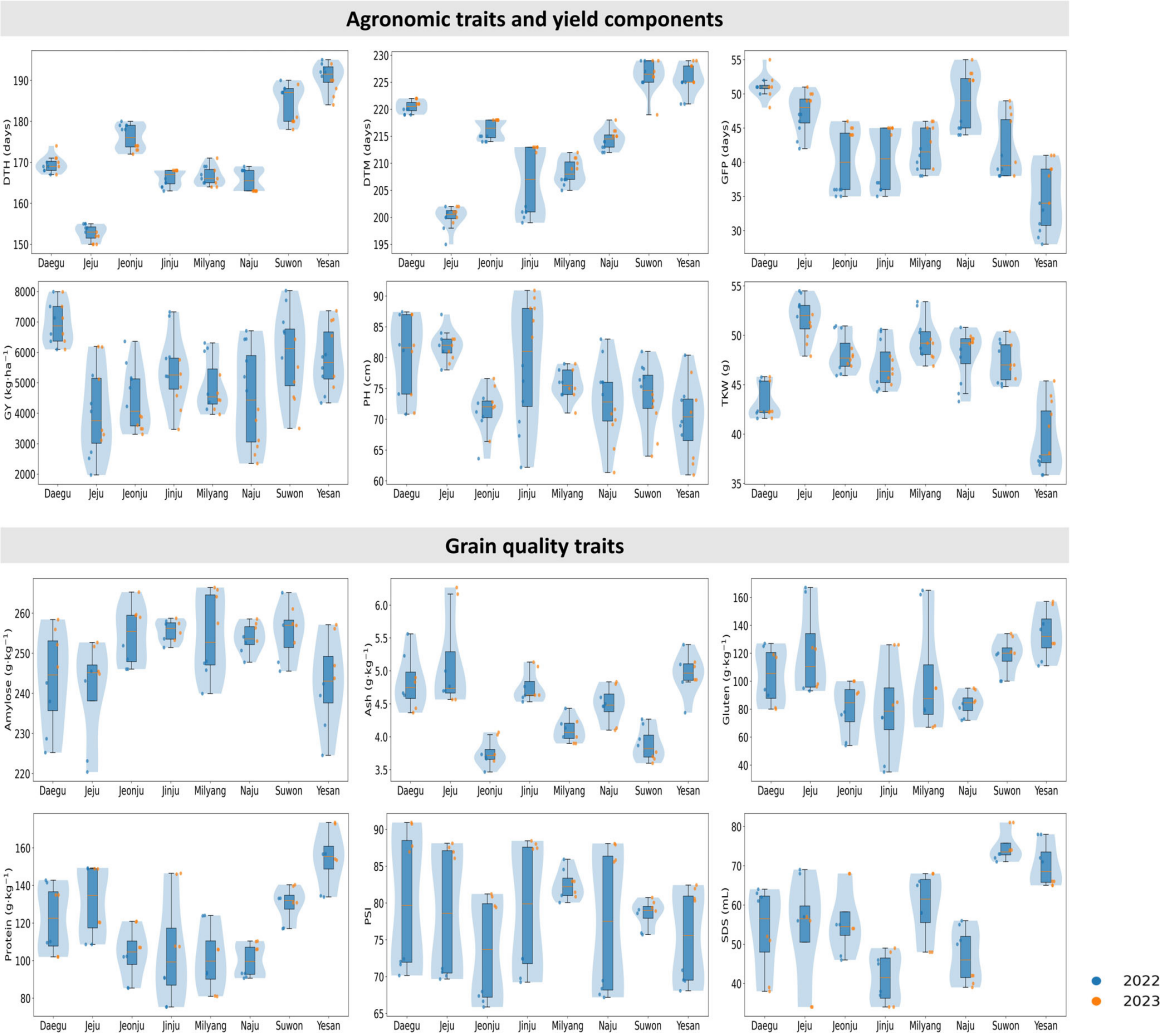
Spatial distribution of agronomic traits, yield components, and grain quality traits across experimental sites, showing spatial variability in agronomic and yield‐related traits (upper panels) and grain quality traits (lower panels).

Temporal and spatial variability in agronomic traits, yield components, and grain quality traits were examined using complete distributional analyses across the 2022 and 2023 growing seasons at eight experimental sites. Figure 5 shows inter‐annual distribution patterns, and Fig. 6 illustrates spatial dispersion among the sites.

Interannual variability was expressed through shifts in central tendency as well as changes in dispersion and distribution shape (Fig. 5). Phenological traits, including DTH and DTM, shifted toward lower values in 2023 than in 2022, indicating a shorter growth duration. In contrast, GFP showed a modest increase in the median values accompanied by reduced dispersion. Grain yield (GY) displayed pronounced interannual differences, with compact distributions in 2022 and broader, right‐skewed distributions with lower central values in 2023. Thousand‐kernel weight exhibited a similar but less pronounced pattern. These temporal distributional shifts indicate that environmental differences between growing seasons influenced not only average trait expression but also the magnitude and consistency of agronomic responses.

Grain quality traits exhibited distinct temporal response patterns (Fig. 5). Protein and gluten content showed consistent upward distributional shifts in 2023, indicating higher overall quality under the climatic conditions of that growing season. In contrast, amylose content exhibited nearly overlapping distributions between years, indicating strong temporal stability. Ash content and SDS sedimentation volume showed moderate interannual variability, whereas the PSI values remained comparatively stable. The contrasting temporal responses of amylose content and protein‐related quality traits further support the existence of trait‐specific environmental sensitivity, with different quality components responding differently to year‐to‐year environmental variation.

Spatial variability across experimental sites was generally more pronounced than inter‐annual variability (Fig. 6). Phenological traits exhibited site‐dependent distributional shifts but remained relatively narrowly distributed across locations. In contrast, yield‐related traits, particularly grain yield and the number of spikes per unit area, exhibited substantial spatial heterogeneity in both range and dispersion, reflecting strong local environmental influences. The greater spatial heterogeneity observed for yield‐related traits suggests that local environmental conditions contribute more strongly to yield formation than to phenological development.

Grain quality traits also exhibited distinct site‐specific patterns (Fig. 6). Protein and gluten content showed clear separation among locations, whereas amylose content exhibited minimal spatial dispersion. Ash content and SDS sedimentation volume showed moderate spatial differentiation, whereas the PSI remained relatively stable across environments. The limited spatial dispersion of amylose content contrasted with the pronounced location‐specific responses of protein‐associated traits, indicating fundamentally different patterns of environmental regulation among grain quality components.

Overall, distribution‐based analyses indicated that environmental effects were expressed not only as shifts in mean trait values but also as changes in variance structure and distributional shape. Temporal effects were primarily associated with systematic distributional shifts between years, whereas spatial effects introduced greater heterogeneity among environments. Together, these results reinforce the distinction between environmentally stable traits, such as phenological traits and amylose content, and environmentally responsive traits, such as yield components and protein‐associated quality traits. This pattern further highlights that environmental regulation of wheat performance is highly trait dependent rather than uniformly expressed across agronomic and grain quality characteristics.

### Climatic structure of wheat production environments

**Figure 7:**
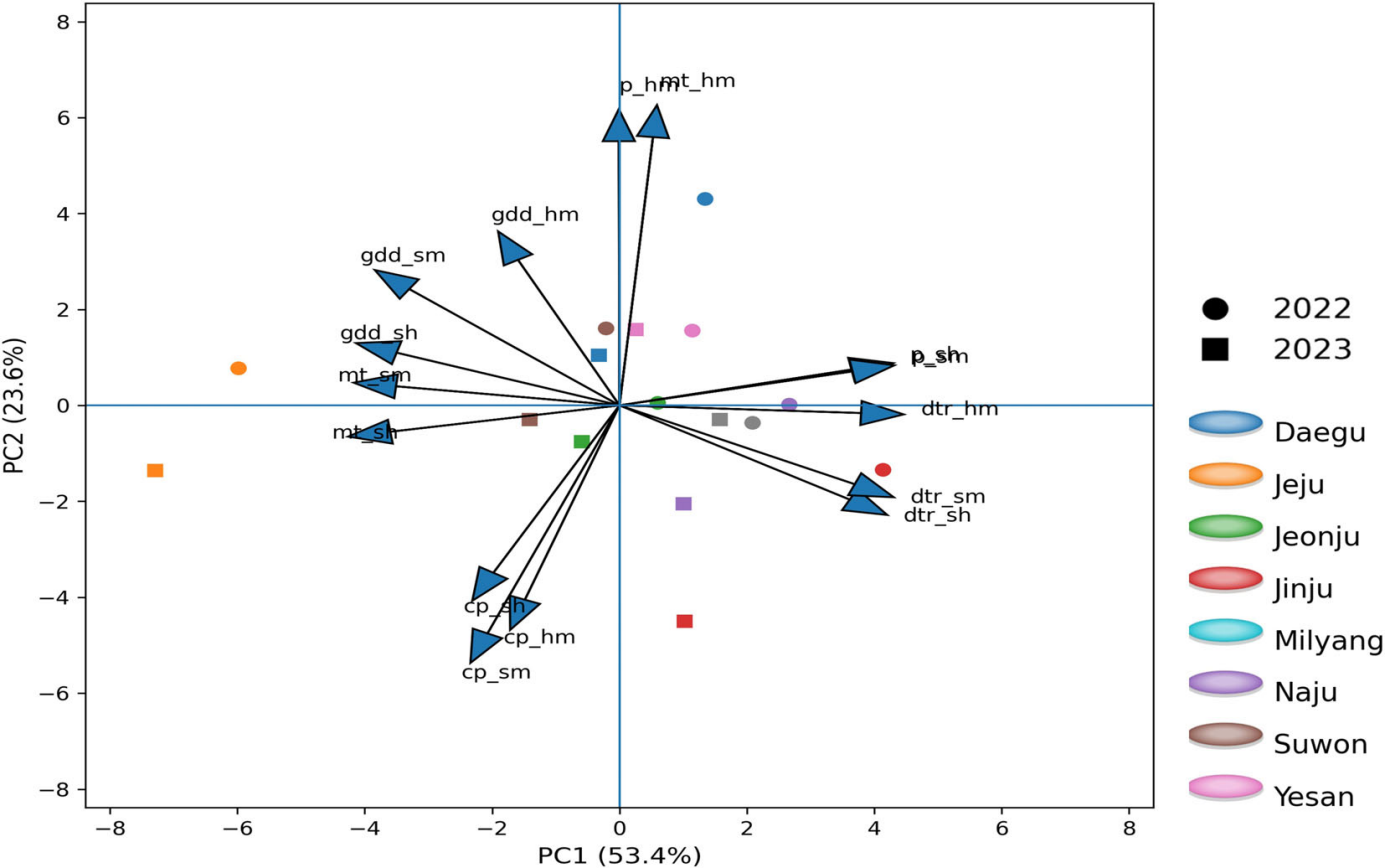
Principal component analysis (PCA) biplot of stage‐specific climatic variables illustrating the climatic structure of wheat production environments across experimental sites and years.

The climatic structure of wheat production environments across sites and years was characterized using PCA based on stage‐specific climatic variables, excluding cumulative solar radiation. The first two principal components (PCs) explained 79.4% of the total climatic variance, with PC1 and PC2 accounting for 53.4% and 23.6%, respectively (Fig. 7). This high explanatory power indicates that environmental heterogeneity across wheat production environments can be effectively summarized using a limited number of dominant climatic gradients, despite substantial variation among sites and years.

PC1 represented the dominant climatic gradient, contrasting environments with greater sunshine duration and diurnal temperature range against those dominated by cumulative thermal and moisture conditions. Sunshine duration during early growth (P_SH) and diurnal temperature range across growth stages loaded positively along PC1, whereas growing degree days, mean temperature, and cumulative precipitation loaded negatively. This gradient therefore represents a broad climatic transition from accumulation‐dominated environments to environments characterized by greater radiative exposure and thermal variability.

PC2 describes a secondary climatic gradient primarily associated with moisture availability across growth stages. Cumulative precipitation exhibited strong positive loadings on PC2, whereas temperature‐ and radiation‐related variables during the heading‐to‐maturity period were associated with the negative direction of the axis, separating wetter environments from relatively drier environments characterized by stronger thermal and radiative inputs during reproductive development. Variation along this gradient was primarily driven by differences in water and energy balance during crop development and grain formation.

Projection of site–year environments into the PCA space revealed clear climatic differentiation among wheat production environments (Fig. 7). Several site–year combinations showed inter‐annual shifts in PCA position, reflecting year‐to‐year changes in the relative balance among radiative, thermal, and moisture conditions. In contrast, other environments maintained relatively stable positions across years, indicating greater climatic consistency. These shifts demonstrate that environmental differences among site–year combinations were not random but reflected systematic variation in coordinated climatic conditions.

Overall, the PCA‐based climatic ordination provided a structured environmental framework for interpreting environmental heterogeneity across production environments. Rather than evaluating individual climatic variables independently, this approach integrated coordinated climatic gradients across multiple developmental stages and condensed complex environmental variation into a small number of biologically meaningful axes. These climatic gradients provide a basis for understanding the contrasting environmental responses observed among wheat traits, including the relatively stable expression of phenological traits and amylose content and the substantially greater environmental sensitivity of yield components and protein‐associated grain quality traits. This framework therefore facilitates the identification of stage‐dependent climatic drivers and trait‐specific patterns of environmental responsiveness across diverse production environments.

The PCA incorporated climatic information across multiple developmental stages to characterize overall environmental structure but grain quality traits are predominantly determined during reproductive growth and grain filling. Table 4 therefore summarizes site‐ and year‐specific climatic conditions during the heading‐to‐maturity period, together with representative grain quality traits to illustrate how the identified climatic gradients are manifested during the critical phase of grain quality formation.

**Table 4:** Site‐ and year‐specific climatic conditions during the heading‐to‐maturity period and representative grain quality traits across wheat production environments

Site	Year	GDD (°C d)	MT (°C)	CP (mm)	P (h)	Protein (g kg^−1^)	Gluten (g kg^−1^)	Amylose (g kg^−1^)
Daegu	2022	1008.7	19.8	40.0	477.6	126.0	109.0	233.6
	2023	937.3	18.3	217.0	360.8	118.5	99.3	253.2
Jeju	2022	812.6	18.1	93.1	356.0	128.8	129.8	233
	2023	845.9	17.0	279.8	324.5	134.6	110.2	248.6
Jeonju	2022	657.2	18.4	55.2	320.8	93.8	66.0	248.1
	2023	823.2	18.4	250.5	309.8	113.9	95.7	264.2
Jinju	2022	608.8	16.8	69.6	329.2	83.1	55.5	255.1
	2023	733.8	16.4	361.6	270.7	126.9	105	256.1
Milyang	2022	713.6	18.0	62.7	325.0	108.6	121.5	245.3
	2023	736.4	16.8	215.1	307.8	93.5	81.2	263.4
Naju	2022	832.4	18.6	46.2	415.2	92.0	77.5	251.3
	2023	924.2	17.5	201.3	344.6	108.1	90.0	256.3
Suwon	2022	739.6	19.0	40.8	384.9	125.1	109.8	253.8
	2023	865.4	19.4	134.7	326.8	135.4	126.8	257
Yesan	2022	568.1	18.4	6.8	348.8	145.4	125.8	234.6
	2023	720.9	18.7	140.8	293.1	163.4	141.5	251

Abbreviations: GDD, growing degree days; MT, mean temperature; CP, cumulative precipitation; P, sunshine duration.

### Climatic regulation of agronomic responses and grain quality

**Figure 8:**
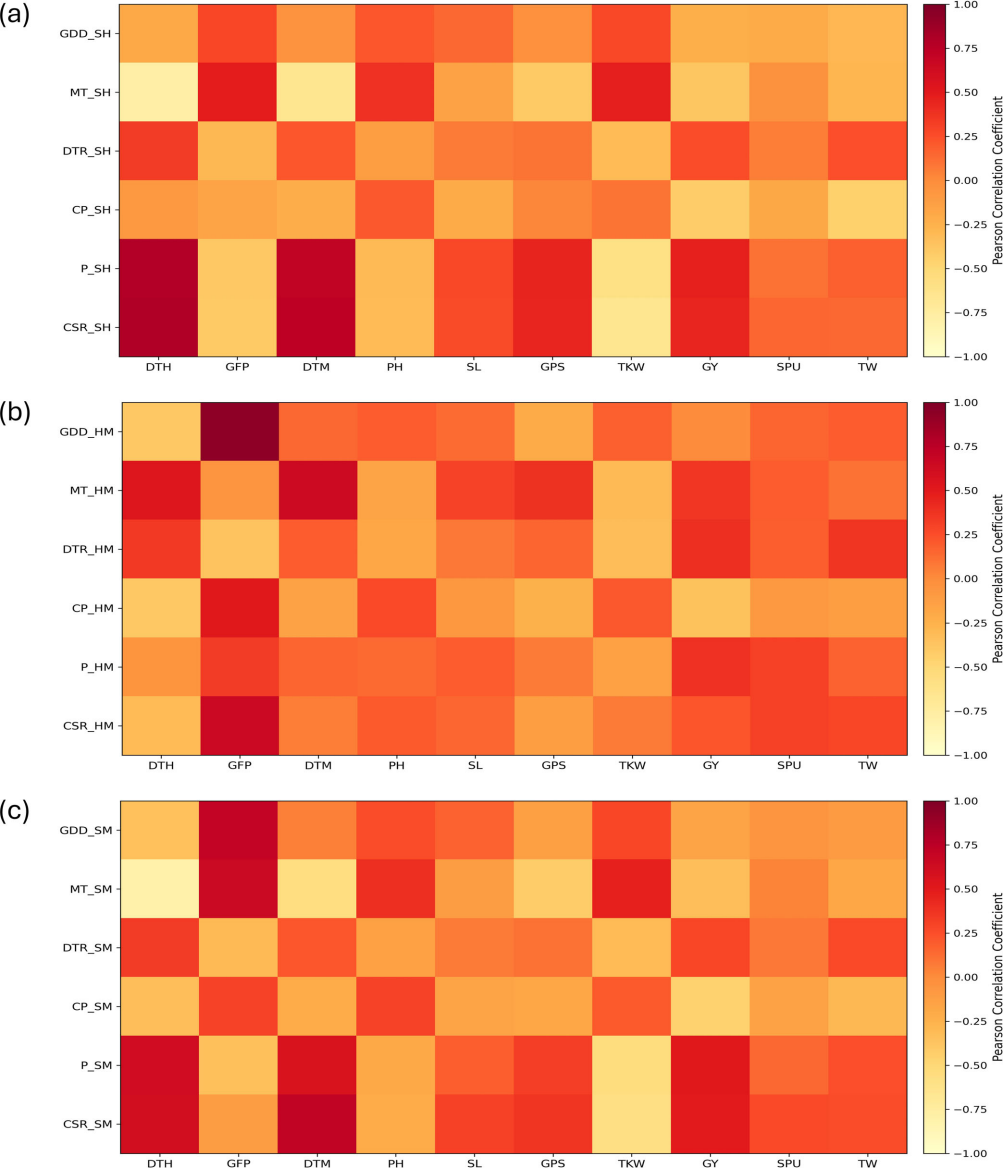
Stage‐specific correlations between climatic variables and agronomic traits across multi‐environment wheat trials, expressed as Pearson correlation coefficients for (a) sowing to heading (SH), (b) heading to maturity (HM), and (c) sowing to maturity (SM).

**Figure 9:**
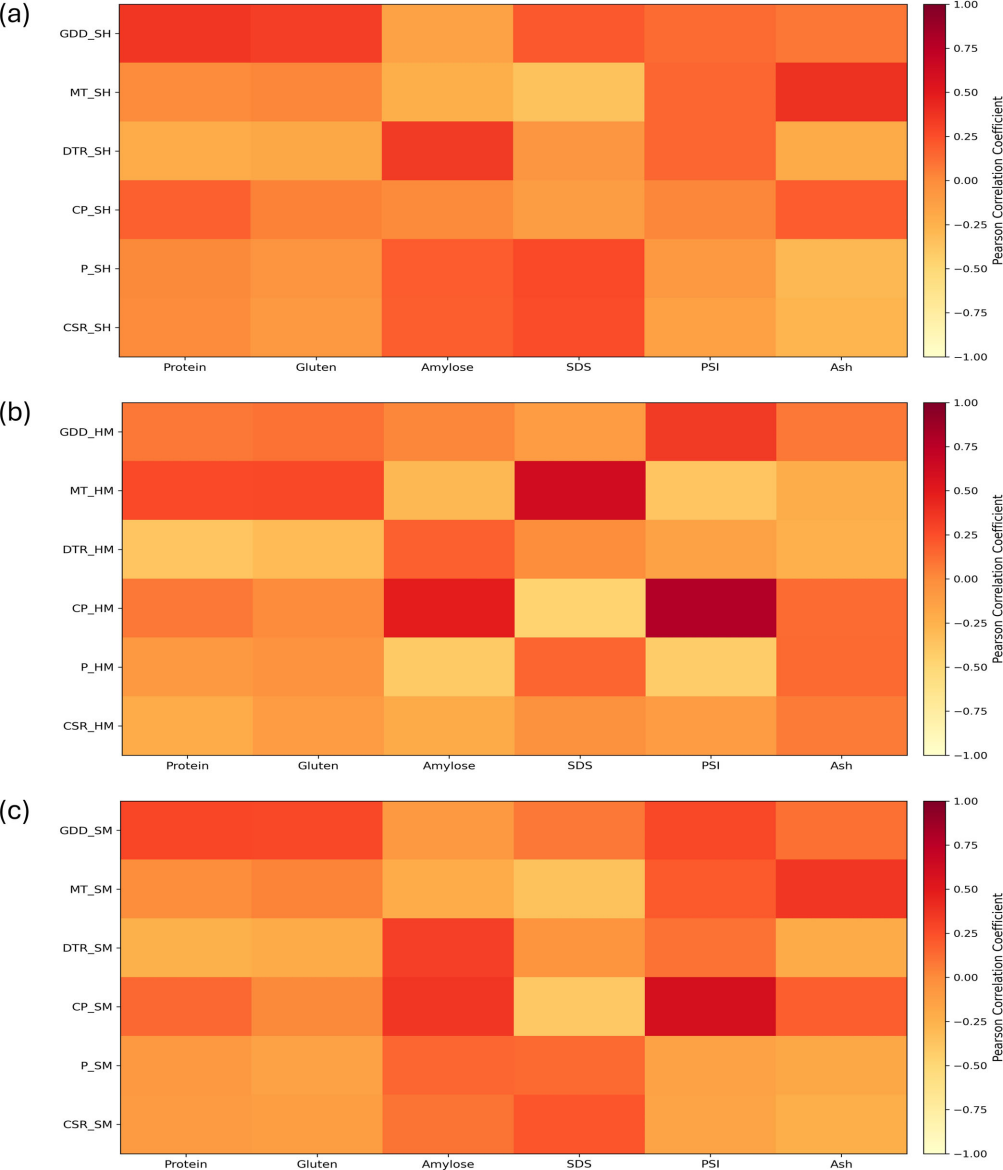
Stage‐specific correlations between climatic variables and grain quality traits across multi‐environment wheat trials, expressed as Pearson correlation coefficients for (a) sowing to heading (SH), (b) heading to maturity (HM), and (c) sowing to maturity (SM).

The relationships between the climatic variables and agronomic and grain quality traits were examined using Pearson correlation analysis to identify developmental stage‐dependent climatic drivers of phenotypic variability (Figs 8 and 9).

Early season climatic conditions were primarily associated with phenological progression and sink establishment (Fig. 8(a)). Thermal accumulation (GDD_SH) was positively correlated with DTH and DTM, indicating that variation in thermal resources during vegetative growth influenced developmental timing. Radiation‐related variables, including sunshine duration (P_SH) and cumulative solar radiation (CSR_SH), were positively associated with spike‐related traits, including spikes per unit area and grains per spike, suggesting a contribution of early season energy availability to reproductive sink development.

In contrast, the diurnal temperature range exhibited weaker and less consistent associations at this stage. These relationships indicate that climatic regulation during early development primarily influenced crop establishment and the formation of yield potential rather than final yield.

Yield‐related traits were most strongly regulated by climatic conditions during reproductive development (Fig. 8(b)). The thermal and radiative variables (GDD_HM, MT_HM, and CSR_HM) showed consistent positive correlations with thousand‐kernel weight and grain yield, emphasizing the importance of mid‐season energy availability for grain filling and yield formation. Precipitation during this period showed relatively weak and variable associations. Compared with the early growth stage, climatic influences during HM showed stronger and more direct relationships with final yield components, indicating that reproductive development represented a critical window of environmental sensitivity for wheat productivity.

When climatic effects were evaluated across the full growing period, the correlations primarily reflected cumulative influences on kernel development and yield performance (Fig. 8(c)). Full‐season thermal indices (GDD_SM and MT_SM) were positively associated with thousand‐kernel weight and grain yield, whereas radiation‐related variables were associated with grain number and kernel weight. The observed patterns indicate that climatic conditions throughout the growing season collectively influenced multiple developmental processes and ultimately determined the expression of yield‐related traits.

The grain quality traits exhibited distinct trait‐dependent climatic responses (Fig. 9). Protein‐related attributes, including protein content, gluten content, and SDS sedimentation volume, were consistently positively associated with thermal accumulation and radiation variables, particularly during the HM and SM periods. These associations demonstrate that protein accumulation and dough‐quality traits were particularly sensitive to climatic conditions during reproductive development and grain filling.

In contrast, amylose content showed the strongest association with the diurnal temperature range rather than with cumulative thermal or radiative variables, indicating sensitivity to temperature variability rather than overall energy accumulation. The PSI and ash content exhibited intermediate and stage‐dependent relationships reflecting more complex patterns of environmental regulation.

The climatic drivers governing grain quality differed substantially among quality components. Whereas protein‐associated traits responded primarily to cumulative thermal and radiative inputs during reproductive development, amylose content was more closely linked to temperature variability. This contrast indicates that grain quality should not be considered as a single environmentally regulated entity but rather as a collection of traits controlled through distinct climatic pathways.

Overall, these results demonstrate that climatic regulation of wheat performance was strongly dependent on both developmental timing and trait type. Early season climatic conditions primarily influenced phenological progression and sink establishment, whereas reproductive‐stage climatic conditions exerted the strongest control over yield formation and protein‐associated grain quality traits. The identification of these stage‐dependent and trait‐specific climatic responses provides a mechanistic explanation for the contrasting stability patterns observed among agronomic and grain quality traits across environments and highlights the existence of multiple climatic pathways governing wheat productivity and grain quality.

### Integrated assessment of grain quality across multi‐environments

**Figure 10:**
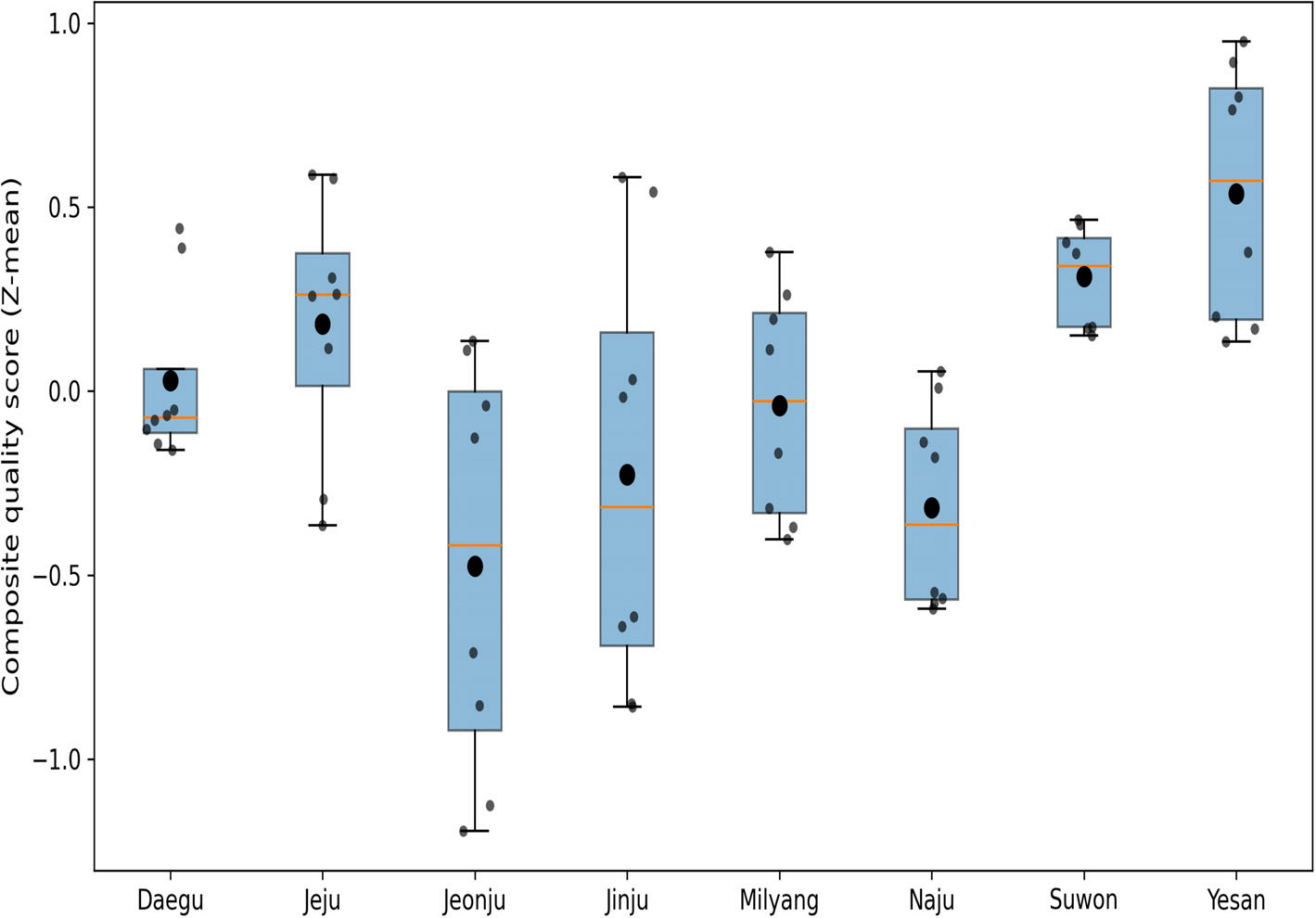
Integrated assessment of wheat grain quality across production environments using a composite grain quality index.

Using the climatic framework defined by the PCA‐based ordination of production environments (Fig. 7), the grain quality index revealed clear differences among environments in both overall grain quality performance and interannual stability (Fig. 10). Environmental differences were characterized by both mean grain quality and the stability of quality expression across years.

Marked differences among environments were evident in both the central tendency and dispersion of the grain quality index values (Fig. 10). Several environments consistently exhibited positive scores, reflecting comparatively high overall grain quality, whereas others were characterized by predominantly negative values, indicating lower composite quality. Environments with similar average index values often differed markedly in the breadth of their distributions, indicating contrasting levels of interannual stability. Narrow distributions reflected consistently favorable or unfavorable grain quality across years, whereas broader distributions indicated greater environmental variability. These results demonstrate that environmental suitability for grain quality cannot be assessed solely on the basis of mean performance because environments with comparable average quality may differ substantially in stability.

The integrated grain quality index complements the trait‐specific analyses by demonstrating how individual climatic responses can collectively determine overall grain quality under different environmental conditions. By combining multiple grain quality traits into a unified framework, the index identifies environments that consistently support high‐quality grain production as well as those in which quality expression is more variable across years. These results show that environmental effects on grain quality were expressed through the combined dimensions of quality level and stability, providing a broader framework for environment‐specific wheat quality assessment under heterogeneous climatic conditions. They also support the concept that grain quality responses are governed by trait‐specific and stage‐dependent climatic pathways that ultimately determine both the magnitude and stability of quality expression across production environments.

## DISCUSSION

Multi‐environmental variability in wheat agronomic performance and grain quality has been widely documented. However, environmental effects have most commonly been interpreted through the use of individual climatic variables or site‐specific comparisons. The present study demonstrated that the environmental regulation of wheat performance can be more coherently understood by integrating heterogeneous climatic conditions into structured climatic gradients and linking these gradients to developmental stage‐specific trait responses. Rather than treating environments as independent site effects, this framework organizes environmental heterogeneity into a coherent climatic space and provides a basis for identifying the mechanisms underlying phenotypic variation across production environments.

The climatic structure identified here integrated radiative conditions, thermal accumulation, temperature variability, and moisture availability into a limited number of dominant gradients. Previous studies have often emphasized the independent effects of temperature or precipitation on wheat yield and quality.^39^, ^40^ In contrast, the present results indicate that phenotypic responses are governed by the balance among multiple climatic components rather than by any single environmental factor. Such multivariate representations of the environmental structure have become increasingly important in genotype–environment interaction studies but remain relatively uncommon in studies that simultaneously evaluate agronomic performance and grain quality. By reducing complex environmental variation into biologically meaningful climatic gradients, the present approach provides a more integrated framework for interpreting environmental influences on wheat production systems.

One of the most important insights emerging from this study is that environmental regulation of wheat performance operates through a hierarchical process linking climatic structure, developmental timing, and trait‐specific sensitivity. Climatic variation among environments was not expressed uniformly across all traits but was filtered through specific developmental stages and physiological processes, resulting in distinct patterns of environmental responsiveness and stability. These patterns were consistently observed across coefficient of variation analyses, distributional analyses, climatic ordination, and stage‐specific climatic correlation analyses.

Phenological traits and amylose content remained comparatively stable despite substantial climatic variation, whereas yield components and protein‐associated quality traits responded strongly to environmental gradients, particularly during reproductive development. Taken together, these results indicate that environmental adaptation in wheat should be understood not simply as differences in mean performance among environments, but as the outcome of coordinated interactions between climatic gradients, critical developmental windows, and trait‐specific regulatory mechanisms.

The climatic analyses revealed that environmental responsiveness was strongly dependent on developmental timing. Early season climatic conditions were primarily associated with phenological progression and sink establishment, whereas climatic conditions during reproductive development exerted the strongest influence on yield formation and protein‐related quality traits. These stage‐dependent responses are consistent with previous reports highlighting the importance of thermal and radiative conditions during spike development, grain set, and grain filling.^41^, ^42^, ^43^, ^44^, ^45^ However, by explicitly separating climatic influences among developmental stages, the present study extended previous approaches based on seasonally averaged climatic indicators – which may have obscured critical windows of environmental sensitivity. The identification of stage‐specific climatic drivers further suggests that distinct climatic pathways regulate different agronomic and grain quality traits.

Grain quality traits exhibited particularly contrasting environmental response patterns. Protein content, gluten content, and SDS sedimentation volume were consistently associated with thermal accumulation and radiative conditions during reproductive development and grain filling. Amylose content exhibited comparatively stable expression across environments and was more strongly associated with temperature variability than with cumulative thermal or radiative inputs. This divergence supports earlier observations that starch composition is under stronger genetic control than protein accumulation,^46^ and demonstrates that grain quality should not be considered a single environmentally regulated entity. Instead, different quality components appear to be governed by distinct climatic pathways and exhibit markedly different environmental sensitivities.

Beyond trait‐specific responses, this study emphasized the importance of environmental effects through both quality level and quality stability. Multi‐environment studies have traditionally focused on the average performance but inter‐annual stability represents a critical dimension of agronomic risk and quality consistency.^47^ Consistent with this perspective, the grain quality index demonstrated that environments with similar mean quality levels often differed substantially in inter‐annual stability. This observation indicates that average quality performance alone may not adequately characterize environmental suitability for wheat quality production. By integrating multiple grain quality traits into a unified framework, the grain quality index revealed that environmental effects are reflected in both overall quality performance and inter‐annual quality stability. This perspective extends recent efforts to incorporate stability metrics into grain quality assessment frameworks and provides a broader basis for environment‐specific evaluation.

The practical implications of these findings are substantial. Traits exhibiting stable expression across environments, such as phenological traits and amylose content, may serve as reliable indicators of cultivar adaptation and broad environmental suitability. In contrast, environmentally responsive traits, including grain yield and protein‐associated quality traits, are more likely to benefit from environment‐specific management strategies and targeted adaptation measures. Consequently, breeding and management programs should recognize that different agronomic and quality objectives may require distinct approaches depending on the environmental sensitivity of the target trait.

This study has several limitations. The analysis was based on a limited number of commercial cultivars and relied on climatic indices aggregated over predefined developmental stages, which may have limited extrapolation to broader genetic backgrounds or short‐term extreme climatic events. However, this stage‐based aggregation was adopted intentionally to align climatic signals with physiological development, thereby enhancing the interpretability of climate–trait relationships. Future studies incorporating a wider range of genotypes, climatic data with higher temporal resolution, and explicit representations of extreme events would provide further information regarding wheat productivity and grain quality responses to increasing climatic variability.

## CONCLUSIONS

This study demonstrated that the environmental regulation of wheat agronomic performance and grain quality is organized through a hierarchical framework in which climatic gradients interact with developmental timing to generate trait‐specific patterns of responsiveness and stability across environments. The integration of multi‐environment field observations with multivariate climatic characterization and developmental stage–specific analyses revealed coordinated climatic pathways governing wheat performance across different phases of crop development.

The effects of climate on wheat were strongly dependent on both developmental timing and trait type. Early season climatic conditions primarily influenced phenological progression and sink establishment, whereas climatic conditions during reproductive growth exerted the strongest control over yield formation and protein‐associated grain quality traits. Amylose content showed comparatively stable expression across environments and distinct climatic responses compared with protein‐related traits, indicating that grain quality components are regulated through different climatic pathways.

A clear distinction emerged between traits exhibiting high stability and those showing strong environmental responsiveness. Phenological traits and amylose content remained relatively consistent across years and locations, whereas yield components and protein‐associated quality traits exhibited substantial environmental sensitivity. These results demonstrate that environmental regulation of wheat performance is highly trait‐dependent and that environmental variability selectively affects specific agronomic and grain quality processes.

Furthermore, the integrated grain quality assessment revealed that environmental differences were expressed not only through variations in overall grain quality level but also through differences in quality stability across years. These patterns indicate that environmental suitability for wheat quality production cannot be adequately evaluated using average quality performance alone and highlight the importance of incorporating stability metrics into multi‐environment quality assessments.

Overall, the analytical framework presented here provides a robust basis for climate‐informed evaluation of wheat productivity and grain quality. By jointly considering climatic structure, developmental timing, and trait stability, this approach offers insights relevant to cultivar evaluation, environment‐specific management, and adaptation of wheat production systems under increasing climatic variability. More broadly, the framework developed here shifts the interpretation of multi‐environment wheat trials from simple comparisons of environmental means toward an integrated understanding of how climatic gradients are translated into stage‐dependent and trait‐specific biological responses.

## FUNDING INFORMATION

This work was supported by the Rural Development Administration, Republic of Korea (Project No. PJ01677103, “Development of New Wheat Variety and Promotion of Breeding Efficiency”), and by the Research Fellowship Program (2026) of the National Institute of Crop Science, Republic of Korea.

## CONFLICT OF INTEREST

The authors declare no conflict of interest related to this study.

## AUTHOR CONTRIBUTIONS

**Goeun Lee:** writing – original draft, data curation. **Jinhee Park:** data curation, investigation. **Kyung‐Min Kim:** methodology, conceptualization. **Chon‐Sik Kang:** resources, conceptualization. **Sookjin Kim:** investigation, resources. **Jeong‐Heui Lee:** project administration, writing – review and editing. **Hyun‐jin Jung:** writing – original draft, writing – review and editing, supervision.

## Data Availability

The data that support the findings of this study are available on request from the corresponding author. The data are not publicly available due to privacy or ethical restrictions.
